# Anti-fibrogenic effect of PPAR-γ agonists in human intestinal myofibroblasts

**DOI:** 10.1186/s12876-017-0627-4

**Published:** 2017-06-07

**Authors:** Jun Bon Koo, Myeong-Ok Nam, Younshin Jung, Jongman Yoo, Duk Hwan Kim, Gwangil Kim, Sung Jae Shin, Kee Myung Lee, Ki Baik Hahm, Jong Woo Kim, Sung Pyo Hong, Kwang Jae Lee, Jun Hwan Yoo

**Affiliations:** 1 Clinical Research Center, CHA Bundang Medical Center, CHA University, Seongnam, South Korea; 2 Department of Microbiology, Institute of Basic Medical Sciences, School of Medicine, CHA University, Seongnam, South Korea; 3 Digestive Disease Center, CHA Bundang Medical Center, CHA University, 59 Yatap-ro, Bundang-gu, Seongnam, 463-712 South Korea; 4 Department of Pathology, CHA Bundang Medical Center, CHA University, Seongnam, South Korea; 5 Department of Gastroenterology, Ajou University School of Medicine, 164, World Cup-ro, Yeongtong-gu, Suwon, 443-380 South Korea; 6 Department of Surgery, CHA Bundang Medical Center, CHA University, Seongnam, South Korea

**Keywords:** Inflammatory bowel disease, Intestinal fibrosis, Myofibroblasts, Troglitazone (TRG), Rosiglitazone (RSG), Extracellular matrix (ECM)

## Abstract

**Background:**

Intestinal fibrosis is a serious complication of inflammatory bowel disease, including Crohn’s disease and ulcerative colitis. There is no specific treatment for intestinal fibrosis. Studies have indicated that peroxisome proliferator-activated receptor- γ (PPAR-γ) agonists have anti-fibrogenic properties in organs besides the gut; however, their effects on human intestinal fibrosis are poorly understood. This study investigated the anti-fibrogenic properties and mechanisms of PPAR-γ agonists on human primary intestinal myofibroblasts (HIFs).

**Methods:**

HIFs were isolated from normal colonic tissue of patients undergoing resection due to colorectal cancer. HIFs were treated with TGF-β1 and co-incubated with or without one of two synthetic PPAR-γ agonists, troglitazone or rosiglitazone. mRNA and protein expression of procollagen1A1, fibronectin, and α-smooth muscle actin were determined by semiquantitative reverse transcription-polymerase chain reaction and Western blot. LY294002 (Akt inhibitor) was used to examine whether Akt phosphorylation was a downstream mechanism of TGF-β1 induced expression of procollagen1A1, fibronectin, and α-smooth muscle actin in HIFs. The irreversible PPAR-γ antagonist GW9662 was used to investigate whether the effect of PPAR-γ agonists was PPAR-γ dependent.

**Results:**

Both PPAR-γ agonists reduced the TGF-β1-induced expression of α-smooth muscle actin which was integrated into stress fibers in HIFs, as determined by actin microfilaments fluorescent staining and α-smooth muscle actin-specific immunocytochemistry. PPAR-γ agonists also inhibited TGF-β1-induced mRNA and protein expressions of procollagen1A1, fibronectin, and α-smooth muscle actin. TGF-β1 stimulation increased phosphorylation of downstream signaling molecules Smad2, Akt, and ERK. TGF-β1 induced synthesis of procollagen1A1, fibronectin, and α-smooth muscle actin through a phosphatidylinositol 3-kinase/Akt-dependent mechanism. PPAR-γ agonists down regulated fibrogenesis, as shown by inhibition of Akt and Smad2 phosphorylation. This anti-fibrogenic effect was PPAR-γ independent.

**Conclusions:**

Troglitazone and rosiglitazone suppress TGF-β1-induced synthesis of procollagen1A1, fibronectin, and α-smooth muscle actin in HIFs and may be useful in treating intestinal fibrosis.

**Electronic supplementary material:**

The online version of this article (doi:10.1186/s12876-017-0627-4) contains supplementary material, which is available to authorized users.

## Background

Intestinal fibrosis is a common complication of inflammatory bowel disease (IBD) occurring in both Crohn’s disease (CD) and ulcerative colitis (UC), but is clinically more apparent in CD [1]. Approximately 75% of CD patients eventually undergo surgery and intestinal strictures represent a main cause of surgery, hospitalization and health care costs for CD patients [2]. Thus, intestinal stricture leads to a significantly impaired quality of life in CD patients [3]. Current anti-inflammatory therapies neither prevent nor reverse the established intestinal fibrosis; thus, the incidence of intestinal strictures in CD has not significantly changed during the last two decades [1]. A recent study showed that intestinal fibrosis, once initiated, is auto-propagative despite the elimination of inflammation, suggesting that the development of direct anti-fibrotic therapy approaches is necessary [4].

Fibrosis is a consequence of local chronic inflammation and is caused by excessive deposition of extracellular matrix (ECM) proteins. ECM proteins including collagen and fibronectin are synthesized by activated myofibroblasts, which are the key effector cells of intestinal fibrosis [5–7]. Abnormal contraction of ECM contributes to tissue distortion and intestinal stricture [8–10]. Activated myofibroblasts express elevated levels of α-smooth muscle actin (α-SMA) and consequently exhibit a markedly enhanced capability to contract ECM [11]. The contractile force of myofibroblasts is generated by stress fibers which are composed of bundles of actin microfilaments (F-actin) [12]. Incorporation of α-SMA into stress fibers enhances the contractile activity of myofibroblasts [13] leading to the formation of specialized contacts with the ECM [14].

The fibrogenic activation of myofibroblasts is controlled by mechanical stress and several cytokines, with the strongest effect elicited by transforming growth factor-beta (TGF-β) [11, 15, 16]. In intestinal myofibroblasts, TGF-β induced ECM and α-SMA expressions are modulated by Smad-dependent and Smad-independent TGF-β signaling pathways [16, 17]. Smad-dependent TGF-β signaling is transduced by phosphorylation of Smad2 and Smad3, which combine with Smad 4 [1, 18, 19]. Smad-independent TGF-β signaling is transduced by phosphorylation of extracellular signal regulated kinase (ERK), c-Jun N-terminal kinase (JNK), p38 mitogen-activated protein kinase (MAPK), Akt, and myosin light chain 2 (Rho signaling) [1, 16, 19–24].

Peroxisome proliferator-activated receptor-γ (PPAR-γ) is a nuclear transcription factor which regulates several cellular functions, including metabolism, adipogenesis, proliferation and differentiation, as well as inflammation [25]. Previous studies have shown that PPAR-γ agonists, such as the thiazolidinedione (TZD) class of anti-diabetic drugs (e.g. troglitazone, rosiglitazone, and pioglitazone) have anti-fibrogenic effect in several body tissues, including the lungs, skin, kidneys, eyes and heart [26–35]. The PPAR-γ agonists inhibit fibrogenesis by regulating the Smad-dependent [27, 30–33] or Smad-independent [23, 29, 31, 36] TGF-β signaling pathways. The mechanism of anti-fibrotic action of PPAR-γ agonists is under investigation but involves both PPAR-γ dependent [26, 32, 37, 38] and PPAR-γ independent [28, 31, 34–36] pathways.

So far, little is known about the anti-fibrotic effect of PPAR-γ agonists on intestinal fibrosis. A previous study found that a novel 5-Aminosalicylic acid (5-ASA) analog with a strong affinity for PPAR-γ has anti-fibrotic properties in intestinal fibrosis [37]. However, the effect of representative synthetic PPAR-γ agonists such as TZD on intestinal fibrosis is still not clear. This study was therefore designed to determine whether PPAR-γ agonists troglitazone (TRG) and rosiglitazone (RSG) have inhibitory effect on the fibrogenic activation of myofibroblasts and which mechanisms are involved.

## Methods

### Reagents

Recombinant human TGF-β1 was obtained from R&D systems (Minneapolis, MN). Troglitazone was purchased from Enzo Life Sciences (Farmingdale, NY). Rosiglitazone and GW9662 were purchased from Cayman Chemical (Ann Arbor, MI). Cell lysis buffer and LY294002 were obtained from Cell Signaling (Danvers, MA). SuperScript™ III reverse transcriptase, Oligo (dT)_12–18_ Primer, dNTP and RNaseOUT™ RNase inhibitor were purchased from Invitrogen (Carlsbad, CA).

### Myofibroblast isolation and culture

Primary HIFs were isolated and cultured with some modifications as previously described [39]. Briefly, HIFs were derived from outgrowths of minced colonic mucosa explants placed on etched polystyrene flasks containing HIFs growth medium consisting of Dulbecco’s modified Eagle’s medium/high glucose (Hyclone, Logan, UT), 10% fetal bovine serum (American Type Culture Collection, Manassas, VA), 4 mmol/L _L_-glutamine (Gibco, Carlsbad, CA), 25 mmol/L HEPES, 100 U/ml penicillin, 100 μg/ml streptomycin, and 0.25 μg/ml amphotericin B (all purchased from Lonza, Walkersville, MD) and used between passage 6 and 10 at 80% confluence. HIFs were isolated from normal colon segments of patients undergoing resection due to colorectal cancer. All normal colon segments were macroscopically confirmed by a pathologist after surgical resection. The project was performed in accordance with the guidelines of the Institutional Review Board of the CHA Bundang Medical Center.

### Cell viability assay (MTT assays)

Thiazolyl blue tetrazolium bromide (MTT; Sigma-Aldrich, Saint Louis, MO) was dissolved in Dulbecco’s phosphate buffered saline (DPBS) at 5 mg/ml. The stock solution was filtered through a syringe filter (pore size, 0.22 μm) and added to the culture medium at a dilution of 1:10. The plates were incubated for 4 h at 37 °C. The culture medium was removed, and the dark brown formazan crystals formed after the reduction of tetrazolium by the mitochondrial dehydrogenises of living cells were dissolved in dimethyl sulfoxide. The optical densities of the samples were measured at a wavelength of 570 nm by plate reader (VersaMax™ Microplate Reader, Molecular Devices, USA). The changes in cell viability after treatment with PPAR-γ agonists were expressed in terms of the control (vehicle treated) cells.

### RNA isolation and semiquantitative RT-PCR

Total RNA was extracted from the HIFs using TRIzol reagent (Ambion, Carlsbad, CA) according to the manufacturer’s instructions. We converted 1 μg of purified total RNA from the samples into cDNA with SuperScript™ III reverse transcriptase and stored all cDNA samples at - 80 °C. The primers used for the PCR are listed in Table 1. The PCR amplification consisted of 35 cycles of 98 °C for 10 s; 60 °C for 10 s; and 72 °C for 30 s. 2 × PCR Master mix solution (AccuPower® PCR PreMix, Bioneer Corporation, South Korea) was used to perform the PCR reactions. The PCR products subjected to electrophoresis on 1% agarose gel were visualized with ethidium bromide. The values for the expression of target genes were normalized against glyceraldehyde-3-phosphate dehydrogenase (GAPDH).

**Table 1:** Information on primers for PCR

Gene	forward primer (5’-3’)	reverse primer (5’-3’)
Collagen1A1	TAGTCTGTCCTGCGTCCTCT	TTATGTTTGGGTCATTTCCA
Fibronectin	CTACGGATGACTCGTGCTTT	TTCCTTCTGCCACTGTTCTC
α-Smooth muscle actin	CTGAGCGTGGCTATTCCTTC	GCTGGAAGGTGGACAGAGAG
GAPDH	AGGTCGGAGTCAACGGATTTGG	ACAGTCTTCTGGGTGGCAGTGATG

### Western blot

HIFs were scraped into ice-cold phosphate buffered saline and harvested by microcentrifugation. The cells were then resuspended in a cell lysis buffer solution [20 mM Tris-HCl (pH 7.5), 150 mM NaCl, 1 mM Na_2_EDTA, 1 mM EGTA, 1% Triton, 2.5 mM sodium pyrophosphate, 1 mM β-glycerophosphate, 1 mM Na_3_VO_4_, and 1 μg/ml leupeptin]. Proteins were resolved on 10% SDS-PAGE and transferred onto PVDF membranes. The membranes were blocked for 1 h with TBST (Tris-buffered saline containing 0.01% Tween 20) containing 5% (w/v) non-fat dry skim milk and incubated for an additional 2 h at room temperature or 24 h at 4 °C with primary antibodies (1:1000 dilution). The antibodies used were as follows: mouse monoclonal procollagen 1A1 (Procol1A1) antibody (1:10; SP1D8, Developmental Studies Hybridoma Bank, Iowa City, IA), mouse monoclonal α-smooth muscle actin (α-SMA) antibody (A2547, Sigma-Aldrich), rabbit polyclonal fibronectin (FN) antibody (ab2413, Abcam, Cambridge, MA), rabbit polyclonal phospho-Akt (Ser473) and Akt antibodies (#9271 and #9272, Cell Signaling), rabbit monoclonal phospho-Smad2 (Ser465/467) antibody (#3108, Cell Signaling), rabbit polyclonal phospho-ERK antibody (#9101, Cell Signaling) and mouse monoclonal β-actin (sc-47,778, Santa Cruz Biotechnology, Dallas, TX). Unbound primary antibodies were removed by three washes with TBST (10 min each). The blots were then incubated with horseradish peroxidase (HRP)-conjugated secondary antibody (1:2000, Santa Cruz Biotechnology) and specific bands were detected by an enhanced chemiluminescence (ECL) detection system (Amersham Pharmacia Biotech, Piscataway, NJ). The signals were captured with a luminescent image analyzer (ChemiDoc™ XRS+ System, Bio-Rad, USA). The quantification of the Western blots was performed using the ImageJ 1.50i software (Wayne Rasband, National Institute of Health, USA).

### Immunocytochemistry

HIFs (1 × 10^4^ cells/well) were seeded onto chamber slides (#30,108, SPL Life Sciences, South Korea) and exposed to TGF-β1 (5 ng/ml) co-incubated with or without TRG/RSG for 24 h, after which the cells were fixed with 4% paraformaldehyde (PFA). After permeabilization with 0.1% Triton-×100 in 1 × PBS and blocking with 5% bovine serum albumin (BSA), the cells were incubated with mouse monoclonal anti-α-SMA antibody (1:500, A2547, Sigma-Aldrich) at 4 °C overnight followed by treatment with goat Alexa488-conjugated anti-mouse IgG antibody (A11029, Thermo Fisher Scientific, Rockford, IL) at room temperature for 2 h. For the staining of actin filaments (F-actin), the cells were incubated with rhodamine-phalloidin (1:200, R415, Invitrogen). After washing, the nuclei were counterstained by Hoechst 33,342 (B2261, Sigma-Aldrich) according to manufacturer’s instructions. The stained sections were immediately covered and examined under a Zeiss LSM510 fluorescence microscope or Zeiss LSM880 confocal laser scanning microscope.

### Statistical analysis

Results are expressed as the means ± the standard deviation (SD) and were analyzed with the Prism statistics software package version 7.0.0 (GraphPad, San Diego, CA). The densitometric data from the western blot assay were analyzed with one-way analysis of variance (ANOVA) with Tukey’s post hoc test. Differences were considered statistically significant at *P* < 0.05.

## Results

### Effect of PPAR-γ agonists on cell viability in human primary intestinal myofibroblasts

**Fig. 1:**
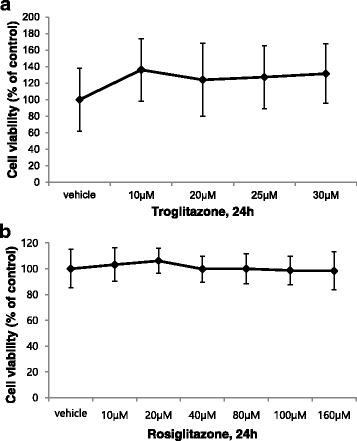
Effect of PPAR-γ agonists on cell viability in human primary intestinal myofibroblasts (HIFs). **a**, **b** HIFs were treated with the indicated concentration of troglitazone (TRG) or rosiglitazone (RSG) for 24 h, after which the cell viability was measured with the MTT assay. The optical density (O.D.) value of the control was regarded as 100%. Results were expressed as a percentage of the basal control (vehicle treatment). All data are expressed as the means ± standard deviation (A: *n* = 8, B: *n* = 10)

To rule out the possibility that the putative effect of PPAR-γ agonists were mediated by cytotoxicity, we performed a cell viability assay after administration of the PPAR-γ agonists (TRG or RSG) in increasing concentrations for 24 h. The effect of TRG (10–30 μM) and RSG (10–160 μM) on HIFs cell viability was assessed by the MTT assay (Figs. 1a and b). Neither TRG nor RSG reduced the cell viability of the HIFs in any of the tested concentrations. Statistical analysis by one-way ANOVA followed by Tukey’s post hoc test showed no significant differences in the cell viability between the vehicle group and the treatment group for each concentration of TRG (10–30 μM) or RSG (10–160 μM).

### Effect of PPAR-γ agonists on TGF-β1-induced expression of α-smooth muscle actin (α-SMA) in human primary intestinal myofibroblasts

**Fig. 2:**
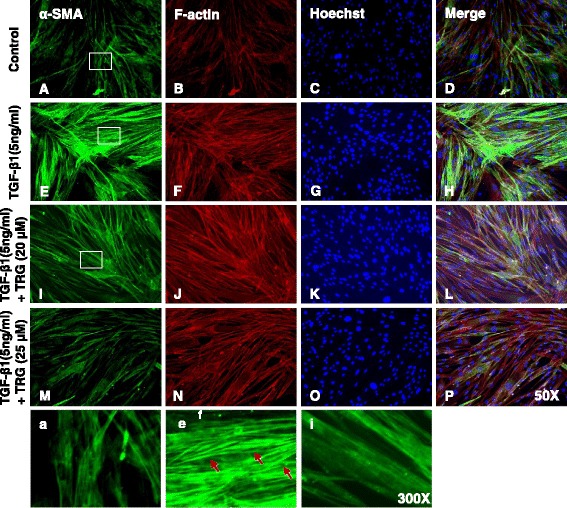
TGF-β1-induced expression of α-smooth muscle actin (α-SMA) is inhibited by troglitazone in human primary intestinal myofibroblasts. HIFs were treated with TGF-β1 (5 ng/ml) co-incubated with or without TRG for 24 h and then stained with α-SMA antibodies and rhodamine-phalloidin counterstained with Hoechst. **a–d** No treatment. **e–h** Treatment with TGF-β1. **i–l** Treatment with TGF-β1 and TRG (20 μM). **m–p** Treatment with TGF-β1 and TRG (25 μM). **a, e**, **i** Enlarged images of the region (within the white box of A, E, and I, each). Red arrows show well-organized stress fibers intensely stained with α-SMA

**Fig. 3:**
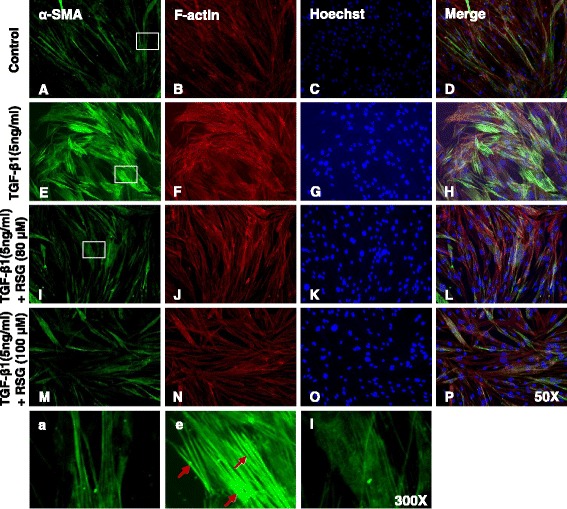
TGF-β1-induced expression of α-smooth muscle actin (α-SMA) is inhibited by rosiglitazone in human primary intestinal myofibroblasts. HIFs were treated with TGF-β1 (5 ng/ml) co-incubated with or without RSG for 24 h and then stained with α-SMA antibodies and rhodamine-phalloidin counterstained with Hoechst. **a–d** No treatment. **e–h** Treatment with TGF-β1. **i–l** Treatment with TGF-β1 and RSG (80 μM). **m–p** Treatment with TGF-β1 and RSG (100 μM). **a, e, i** Enlarged images of the region (within the white box of *A*, *E*, and *I*, each). Red arrows show well-organized stress fibers intensely stained with α-SMA

To determine whether PPAR-γ agonists inhibit fibrogenic activation of myofibroblasts, HIFs were treated with an increasing concentration of TRG or RSG and simultaneously stimulated with TGF-β1. As shown in Figs. 2a and 3a, the HIFs in the control group expressed a basal level of α-SMA. TGF-β1 induced a significant increase in α-SMA expression showing fibrogenic activation of the HIFs (Figs. 2e and 3e). Treatment with TRG (Figs. 2i and m) or RSG (Figs. 3i and m) inhibited the TGF-β1 induced increase of α-SMA expression, which is also demonstrated in a large field (Additional file 1: Figure S1 and Additional file 2: Figure S2).

Increased α-SMA expression and the subsequent assembly of α-SMA into stress fibers are hallmarks of activated fibrogenic myofibroblasts [40]. In TGF-β1-stimulated cells, α-SMA staining showed well-organized intensely stained stress fibers (Figs. 2e and 3e). In contrast, cells stimulated with TGF-β1 in the presence of TRG or RSG exhibited a diffuse, muted α-SMA staining with a lack of organized stress fibers (Figs. 2i and 3i) similar to the α-SMA staining observed in the control (Figs. 2a and 3a).

To determine whether PPAR-γ agonists affect the stress fiber formation in HIFs, we stained the stress fibers with rhodamine-phalloidin by immunocytochemistry. When compared with the controls, TGF-β1 increased the assembly of stress fibers (Figs. 2f and 3f). However, neither TRG nor RSG significantly reduced the stress fiber formation in the HIFs (Figs. 2j, n, 3j and n).

In summary, these immunocytochemistry data suggest that PPAR-γ agonists inhibit TGF-β1-induced α-SMA expression but not the stress fiber formation in HIFs.

### PPAR-γ agonists inhibit TGF-β1-induced ECM and α-SMA expression in human intestinal myofibroblasts

**Fig. 4:**
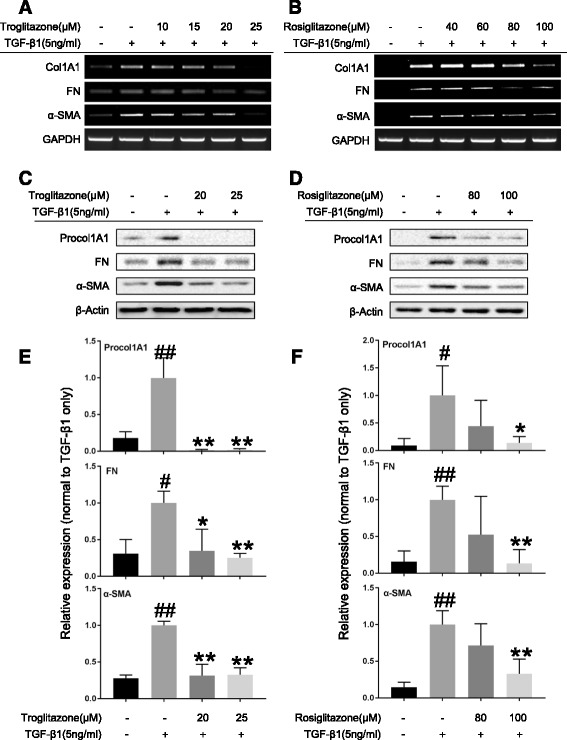
PPAR-γ agonists inhibit TGF-β1-induced fibrogenic activation of human intestinal myofibroblasts. **a–f** HIFs were treated with TGF-β1 (5 ng/ml) co-incubated with or without PPAR-γ agonists (*A*, *C*, *E*: TRG; *B*, *D*, *F*: RSG) for 24 h. **a**, **b** Representative RT-PCRs show the mRNA expression of collagen1A1 (Col1A1), fibronectin (FN), and α-SMA, with GAPDH as the endogenous control. **c**, **d** Representative Western blots show the protein expression of procollagen1A1 (Procol1A1), FN, and α-SMA, with β-Actin as the endogenous control. **e**, **f** Plots of relative protein expression of Procol1A1, FN, and α-SMA normalized to densitometric values obtained in cells stimulated with only 5 ng/ml TGF-β1. Data are the means ± SD averaged for three (E) or four (F) independent experiments. ^#^ *P* < 0.05 versus control group; ^##^ *P* < 0.01 versus control group; *P < 0.05 versus TGF-β1 only group; **P < 0.01 versus TGF-β1 only group. Data were analyzed by one-way ANOVA followed by Tukey’s post hoc test

As shown in Figs. 4a and b, TGF-β1 markedly increased the mRNA expression of collagen1A1, FN, and α-SMA in the HIFs. TRG and RSG abolished this effect in a concentration-dependent manner. The anti-fibrotic effect of TRG and RSG was also identified at the protein level. As shown in Figs. 4c-f, the TGF-β1-induced upregulation in protein expression of Procol1A1, FN and α-SMA was significantly reduced by TRG and RSG. These data suggest that the PPAR-γ agonists inhibit TGF-β1-induced fibrogenic activation of HIFs by downregulating ECM and α-SMA at both the mRNA and protein levels.

### TGF-β1 induces ECM and α-SMA expression by phosphorylation of Akt in human intestinal myofibroblasts

**Fig. 5:**
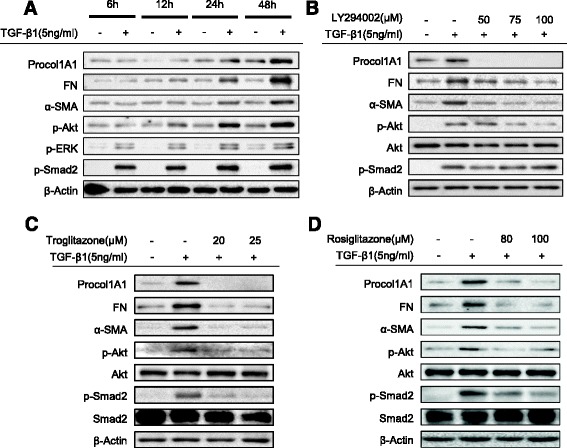
The mechanism for the anti-fibrogenic effect of PPAR-γ agonists on TGF-β1-induced fibrogenic activation in human primary intestinal myofibroblasts. **a** The effect of TGF-β1 stimulation on phosphorylation of signaling molecules in HIFs (Western blot). **b** The effect of Akt inhibitor (LY294002) on TGF-β1 induced expression of Procol1A1, FN, and α-SMA in HIFs (24 h, Western blot). **c**, **d** Effect of PPARγ agonists on TGF-β1 induced phosphorylation of Akt and Smad2 in HIFs (24 h, Western blot)

Previous evidence suggests that TGF-β induced fibrogenesis in several body tissues occurs through Smad-dependent (phosphorylation of Smad2 or Smad3) or Smad-independent (phosphorylation of ERK or Akt) mechanisms [1, 19, 22, 23]. We first tested which signaling molecules were increased by incubating TGF-β1. As summarized in Fig. 5a, the phosphorylation of Akt was observed starting at 12 h after TGF-β1 stimulation and persisting for at least 48 h. Phosphorylation of ERK and Smad2 were also observed with incubation of TGF-β1 at 6 h and persisting for at least 48 h. Expressions of Procol1A1, FN, and α-SMA started to increase at 12 h after the TGF-β1 treatment. PI3 kinase/Akt pathway activation by TGF-β1 has been shown to be crucial in TGF-β1 induced fibrogenesis in several tissues including intestine [21, 23, 36, 41, 42].

To test whether phosphorylation of Akt was a key signal for TGF-β1 induced ECM and α-SMA expression in HIFs, we used a PI3 kinase inhibitor (LY294002). As expected, LY294002 diminished the TGF-β1-induced increase in Procol1A1, FN, and α-SMA expression in a dose dependent manner (Fig. 5b). This result indicates that the TGF-β1 stimulated ECM and α-SMA expression occurs through the PI3K/Akt pathway. However, 50 μM of LY294002, a dose which was sufficient to inhibit the TGF-β1-induced expression of Procol1A1, FN, and α-SMA, did not block the Akt phosphorylation. This suggests that Akt phosphorylation alone does not appear to be sufficient to induce the Procol1A1, FN, and α-SMA.

We also assessed whether phosphorylated Smad2 expression was affected by LY294002; however, there was no significant change (Fig. 5b). This result suggests that Akt phosphorylation is not required for Smad2 phosphorylation in TGF-β1 induced ECM and α-SMA expression.

### PPAR-γ agonists decrease TGF-β1-induced phosphorylation of Akt and Smad2

**Fig. 6:**
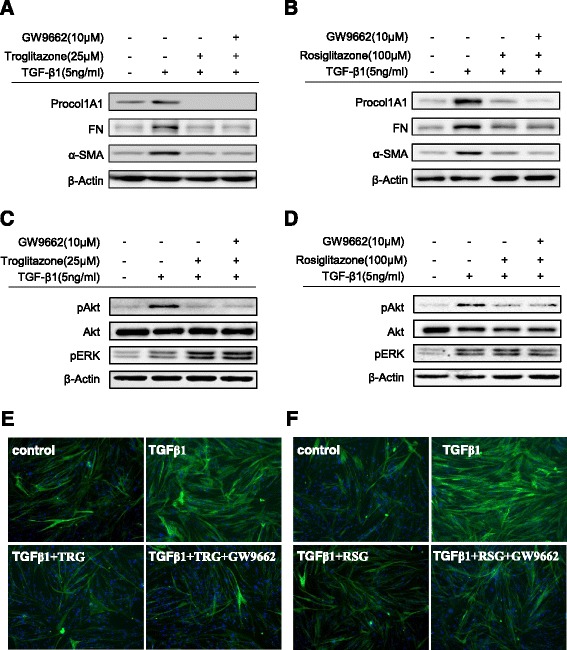
The anti-fibrogenic effect of PPAR-γ agonists is PPAR-γ independent. **a–d** HIFs were pretreated with GW9662, followed by TGF-β1 (5 ng/ml) with or without PPAR-γ agonists (*A*, *C*: TRG; *B*, *D*: RSG). Representative Western blots show protein expression of Procol1A1, FN, α-SMA, pAkt, and pERK with β-Actin as the endogenous control. **e**, **f** HIFs on chamber slides were treated with TGF-β1 (5 ng/ml), TRG (25 μM, Fig. 6e) or RSG (100 μM, Fig. 6f), and GW9662 (10 μM) for 24 h and then stained with α-SMA antibodies and counterstained with Hoechst

Our next question was whether PPAR-γ agonists blocked the TGF-β1-induced increases in Procol1A1, FN, and α-SMA expression by decreasing the phosphorylation of Akt. As shown in Figs. 5c and d, TRG and RSG decreased Akt phosphorylation stimulated by TGF-β1. Moreover, both PPAR-γ agonists decreased Smad2 phosphorylation induced by TGF-β1 in a dose dependent manner. To evaluate whether inhibition of ERK phosphorylation is also involved in the anti-fibrotic mechanism of the PPAR-γ agonists, we checked ERK phosphorylation with a similar experiment design. However, interestingly, the PPAR-γ agonists did not affect ERK phosphorylation (Figs. 6c and d).

### Suppression of ECM, α-SMA, and phosphorylated Akt (pAkt) expression by PPAR-γ agonists are PPAR-γ independent

To further investigate the PPAR-γ dependence of the anti-fibrogenic effect of the PPAR-γ agonists, a highly specific and irreversible PPAR-γ antagonist (GW9662) was used to block the activity of PPAR-γ. HIFs were pretreated with or without GW9662 for 30 min prior to the addition of TRG or RSG co-incubated with TGF-β1. As shown in Figs. 6a and b, GW9662 was unable to prevent the down-regulation of Procol1A1, FN, or α-SMA expression induced by the PPAR-γ agonists suggesting that the anti-fibrogenic actions of the PPAR-γ agonists likely occurred through PPAR-γ independent mechanisms. Furthermore, we identified that the attenuated pAkt expression by the PPAR-γ agonists was not reversed by GW9662 (Figs. 6c and d). This result shows that the attenuation of Akt phosphorylation by the PPAR-γ agonists is also PPAR-γ independent. We confirmed the PPAR-γ independent mechanism by α-SMA immunocytochemistry. As shown in Figs. 6e (TRG) and 6 F (RSG), the GW9662 failed to reverse the inhibition of α-SMA expression by TRG or RSG.

## Discussion

PPAR-γ agonists are emerging as potential therapeutics for fibrotic diseases in multiple tissues outside of the intestine [26, 27, 36, 43]. Several natural and synthetic PPAR-γ ligands are known to activate PPAR-γ, such as prostaglandins, thiazolidinediones (TRG and RSG), and 5-ASA [37]. A recent study showed the anti-fibrotic action of a new PPAR-γ modulator, which belongs to the 5-ASA analogs, in intestinal fibrosis [37]. However, the anti-fibrotic action and mechanism of other typical PPAR-γ agonists on human intestinal fibrosis remain poorly understood.

In the present study, we have identified for the first time that two synthetic PPAR-γ agonists (TRG and RSG) inhibit the TGF-β1-induced fibrogenic activation of HIFs by reducing the expression of ECM (Procol1A1 and FN) and α-SMA. In addition, we found that TGF-β1 induces ECM and α-SMA expression by activating the PI3 kinase/Akt pathway as a Smad-independent TGF-β signaling mechanism. Finally, we showed that PPAR-γ agonists suppress TGF-β1-induced synthesis of ECM and α-SMA in HIFs by blocking Akt phosphorylation.

We investigated whether PPAR-γ agonists regulate the fibrogenic activation of HIFs from two different perspectives. One was a change in the ECM molecule (Procol1A1 and FN) expression as a ‘final product of fibrogenesis’ and the other was a change in the α-SMA expression as a marker showing ‘contractile force’ [13]. Therefore, an increase in the Procol1A1, FN, and α-SMA expression indicates the fibrogenic activation of myofibroblasts. By evaluating the mRNA and protein expression levels of Procol1A1, FN, and α-SMA, we showed that PPAR-γ agonists significantly inhibited the TGF-β1-induced fibrogenic activation of HIFs. This is consistent with previous reports in which several synthetic PPAR-γ agonists suppressed TGF-β-induced collagen, FN, and α-SMA expression in a variety of tissues [26, 27, 36]. In addition to α-SMA, formation of stress fibers in myofibroblasts exerts a contractile activity, and TGF-β1 also enhances the stress fiber formation and contractile force in myofibroblasts [44]. In corneal keratocytes, pioglitazone decreased TGF-β induced stress fiber formation, as determined by decreased F-actin fluorescent signal [45]. However, in our study, TRG and RSG failed to reduce stress fiber formation. Further molecular studies should be performed to confirm the effect of PPAR-γ agonists on stress fiber formation.

In the present study, we showed that TGF-β1-mediated PI3 kinase/Akt activation may be important in the fibrogenic activation (ECM and α-SMA expression) of HIFs. However, the phosphorylated Akt alone does not appear to be sufficient to induce the fibrogenesis. Another downstream target of PI3 kinase pathway, PAK2 (p21-activated kinase 2) has been demonstrated to mediate fibrogenesis induced by TGF-β, via activation of c-Abl (Albelson kinase) [46, 47]. Therefore, further studies should elucidate whether the PI3 kinase/PAK2 pathway also plays a role in the intestinal fibrosis and is down-regulated by PPAR-γ agonists. In intestinal myofibroblasts, TGF-β induced fibrogenic activation is regulated by Smad-dependent and Smad-independent TGF-β signaling [16, 17]. Phosphorylation and nuclear translocation of Smad2/3 are required in Smad-dependent TGF-β signaling [1, 20]. Increased phosphorylated Smad2/3 expression observed in intestinal stricture in CD also supports the fibrogenic role of the Smad-dependent TGF-β pathway [48]. Consistent with previous reports, we found that TGF-β1 increased the phosphorylation of Smad2 as well as the ECM and α-SMA expression in HIFs.

Furthermore, we identified a Smad-independent TGF-β signaling pathway through PI3 kinase/Akt that is involved in TGF-β induced ECM and α-SMA expression in HIFs. In several tissues including the intestines [20, 21, 23, 36, 41, 42, 49], the PI3 kinase/Akt pathway has been proposed to be one of the Smad-independent TGF-β signaling cascades. In our study, TGF-β1-stimulated Smad2 phosphorylation in HIFs was insensitive to a PI3 kinase inhibitor (Akt inhibitor; LY294002), suggesting that TGF-β1-stimulated Smad2 phosphorylation occurs independently of the PI3 kinase/Akt pathway, which is similar to previous reports [50, 51]. In other words, PI3 kinase/Akt activation may contribute to the TGF-β induced fibrogenic activation of HIFs in a Smad independent manner. However, we did not investigate whether the nuclear translocation or transcriptional activity of the phosphorylated Smad2 are sensitive to the PI3 kinase inhibitor. Although a recent study has shown that in fibroblasts, PI3 kinase activation in response to TGF-β does not affect Smad2/3 phosphorylation, nuclear translocation, and transcriptional activity [51], the possibility that the nuclear translocation or binding activity of phosphorylated Smad2 are sensitive to the PI3 kinase inhibitor in HIFs still cannot be excluded.

In agreement with other studies in different tissues [23, 29, 36], we found that synthetic PPAR-γ agonists exert an anti-fibrogenic effect in HIFs by attenuating the Akt activation induced by TGF-β1. The reduced Akt phosphorylation by the PPAR-γ agonists was not reversed by GW9662 pretreatment. Taken together with our results that GW9662 did not prevent the decrease in the ECM and α-SMA expression by the PPAR-γ agonists, this result supports that the anti-fibrogenic action of the PPAR-γ agonists in HIFs occurs through a PPAR-γ independent mechanism. Furthermore, we identified that PPAR-γ agonists reduced Smad2 phosphorylation in a dose dependent manner. This finding is consistent with previous reports that PPAR-γ agonists attenuate the pro-fibrotic responses induced by TGF-β by blocking the phosphorylation of Smad2/3 [27, 30–33]. Taken together, TRG and RSG inhibit the fibrogenic activation of HIFs mediated by TGF-β1 through the suppression of the Smad-dependent and Smad-independent pathways.

Previous studies have shown that the anti-fibrogenic effect of PPAR-γ agonists can be mediated through both PPAR-γ-dependent [26, 32, 37, 52] and PPAR-γ-independent pathways [23, 28, 31, 34–36]. For example, in keloid fibroblasts, the inhibitory effect of TRG on TGF-β1-stimulated collagen type I expression was PPAR-γ-dependent because the PPAR-γ antagonist (GW9662) completely reversed the inhibitory effect [32]. However, in corneal myofibroblasts, the mechanism through which TRG and RSG inhibited fibrosis has occurred by downregulating p38 mitogen-activated protein kinase (MAPK) phosphorylation through a PPAR-γ independent manner, because neither pharmacologic inhibition of PPAR-γ (GW9662) nor transfection with a dominant negative PPAR-γ mutant blocked the anti-fibrotic properties of TRG and RSG to decrease collagen type I, FN, and α-SMA expression [28]. A prior work using intestinal myofibroblasts suggested that a novel PPAR-γ agonist (5-ASA analog) was able to inhibit TGF-β1-induced FN and α-SMA expression in a PPAR-γ-dependent manner [37]. However, in our work, TRG and RSG inhibited TGF-β1-induced Procol1A1, FN, and α-SMA in a PPAR-γ-independent manner. Why our findings are different from those of Speca et al [37] remains a matter of speculation. Differences in cell types (primary cells in our study versus cell lines in theirs), treatment times (1 day in our study versus 4 days in theirs), and the mechanisms of action of a novel PPAR-γ agonist (thiazolidinediones tested here versus 5-ASA analog in theirs) are among the possible explanations.

## Conclusions

**Fig. 7:**
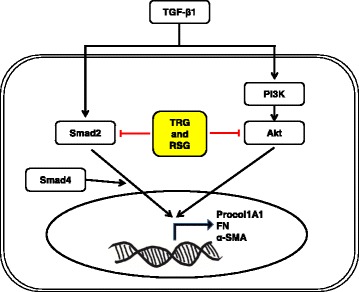
A proposed model showing the mechanism of action of PPAR-γ agonists (TRG and RSG) on TGF-β1-induced fibrogenic activation in human primary intestinal myofibroblasts. TGF-β1 induces expression of Procol1A1, FN, and α-SMA by activating the PI3K/Akt pathway and Smad2. PPARγ agonists (TRG and RSG) inhibit TGF-β-induced fibrogenic responses by blocking Akt signaling and Smad2 phosphorylation

Based on our observations, we propose a model (Fig. 7) depicting the mechanism of action for the PPAR-γ agonists (TRG and RSG) on TGF-β1-induced fibrogenic activation of human primary intestinal myofibroblasts. TGF-β1 stimulates the expression of ECM and α-SMA by activating the PI3K/Akt pathway and Smad2. PPAR-γ agonists (TRG and RSG) inhibit the TGF-β1-induced fibrogenic responses by blocking Akt signaling and Smad2 phosphorylation. In summary, our report provides novel evidence for an anti-fibrogenic effect from synthetic PPARγ agonists in human intestinal myofibroblasts. However, future studies should elucidate downstream and upstream components of the Akt pathway affected by synthetic PPARγ agonists and in vivo experiments should be performed. Nevertheless, our results suggest that the synthetic PPAR-γ agonists, TRG and RSG, may be a promising class of anti-fibrotic drugs for intestinal applications.

## Additional files

Additional file 1: Figure S1. Effect of troglitazone (TRG) on TGF-β1-induced expression of α-smooth muscle actin (α-SMA) in human primary intestinal myofibroblasts (HIFs). HIFs on chamber slides were treated with TGF-β1 (5 ng/ml), TRG (20 and 25 μM) for 24 h and then stained with α-SMA antibodies and counterstained with Hoechst. (PPTX 1065 kb)

Additional file 2: Figure S2. Effect of rosiglitazone (RSG) on TGF-β1-induced expression of α-smooth muscle actin (α-SMA) in human primary intestinal myofibroblasts (HIFs). HIFs on chamber slides were treated with TGF-β1 (5 ng/ml), RSG (80 and 100 μM) for 24 h and then stained with α-SMA antibodies and counterstained with Hoechst. (PPTX 1146 kb)

## Abbreviations

5-ASA: 5-Aminosalicylic acid
ANOVA: Analysis of variance
CD: Crohn’s disease
ECM: Extracellular matrix
ERK: Extracellular signal regulated kinase
FN: Fibronectin
GAPDH: Glyceraldehyde-3-phosphate dehydrogenase
HIFs: Human primary intestinal myofibroblasts
IBD: Inflammatory bowel disease
JNK: C-Jun N-terminal kinase
MAPK: Mitogen-activated protein kinase
PPAR-γ: Peroxisome proliferator-activated receptor- γ
Procol1A1: Procollagen1a1
RSG: Rosiglitazone
SD: Standard deviation
TGF-β: Transforming growth factor-beta
TRG: Troglitazone
TZD: Thiazolidinedione
UC: Ulcerative colitis
α-SMA: α-smooth muscle actin
