# Supplementary figures and images for: Anti-fibrogenic effect of PPAR-γ agonists in human intestinal myofibroblasts
Source: BMC Gastroenterol. 2017 Jun 7;17:73. doi: 10.1186/s12876-017-0627-4 (PMC5463383; doi:10.1186/s12876-017-0627-4)

## Slide 1
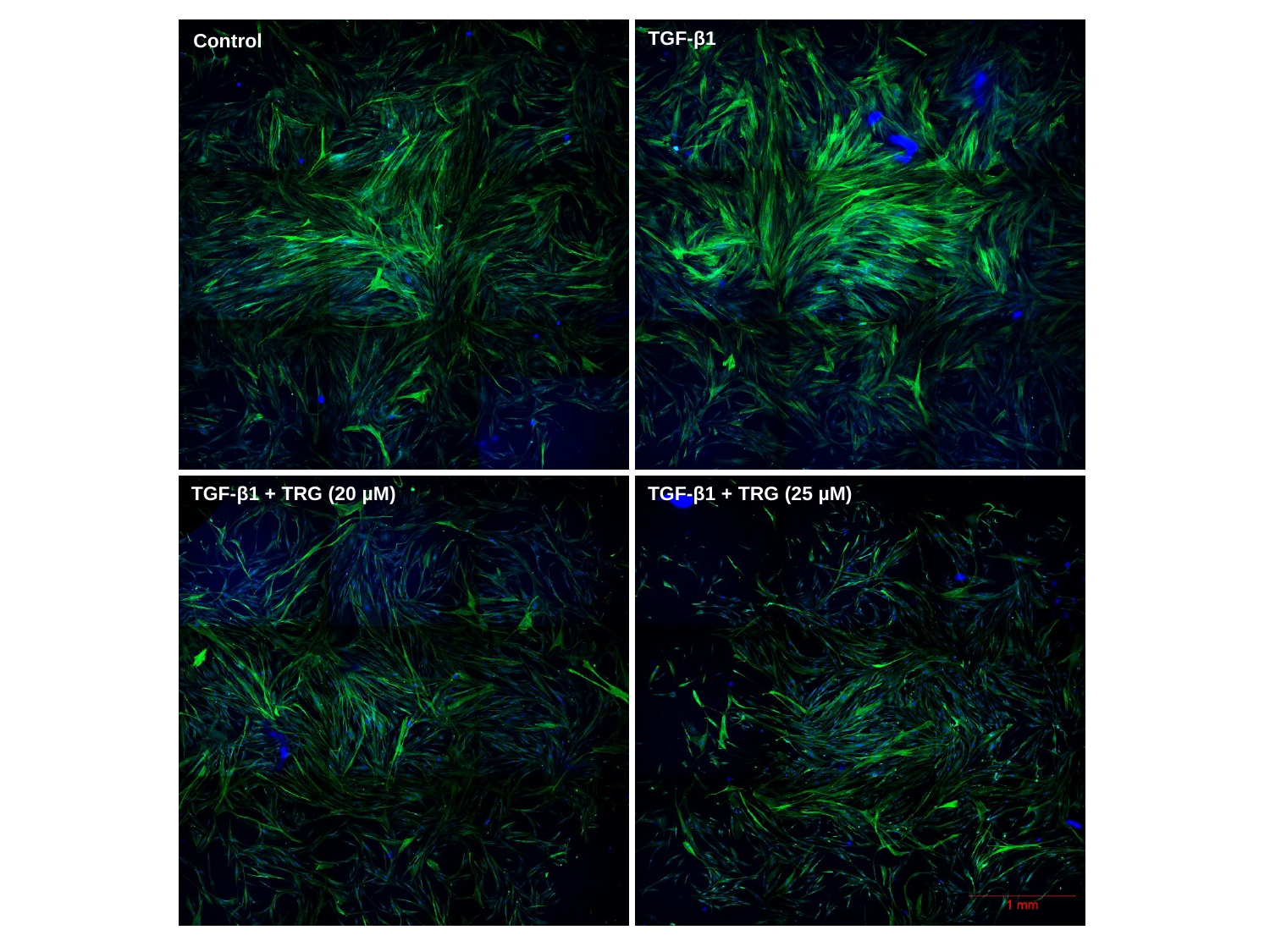

TGF-β1
Control
TGF-β1 + TRG (20 µM)
TGF-β1 + TRG (25 µM)

Supplement: Supplementary file 1 — Effect of troglitazone (TRG) on TGF-β1-induced expression of α-smooth muscle actin (α-SMA) in human primary intestinal myofibroblasts (HIFs). HIFs on chamber slides were treated with TGF-β1 (5 ng/ml), TRG (20 and 25 μM) for 24 h and then stained with α-SMA antibodies and counterstained with Hoechst. (PPTX 1065 kb) [file 12876_2017_627_MOESM1_ESM.pptx]

## Slide 1
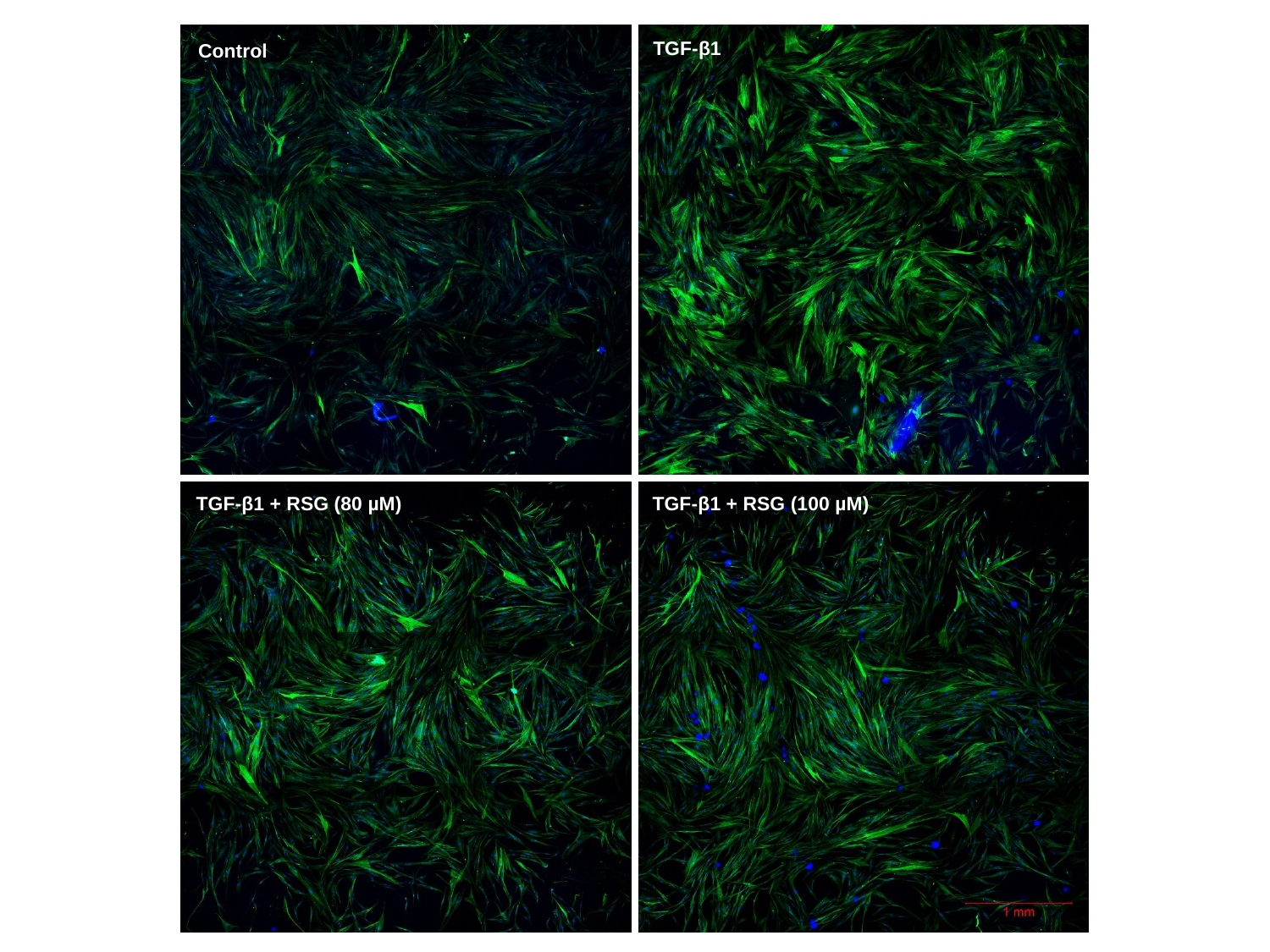

TGF-β1
Control
TGF-β1 + RSG (80 µM)
TGF-β1 + RSG (100 µM)

Supplement: Supplementary file 2 — Effect of rosiglitazone (RSG) on TGF-β1-induced expression of α-smooth muscle actin (α-SMA) in human primary intestinal myofibroblasts (HIFs). HIFs on chamber slides were treated with TGF-β1 (5 ng/ml), RSG (80 and 100 μM) for 24 h and then stained with α-SMA antibodies and counterstained with Hoechst. (PPTX 1146 kb) [file 12876_2017_627_MOESM2_ESM.pptx]
